# Causes and Management Outcome of Small Intestinal Obstruction in Nekemte Referral Hospital, Nekemte, Ethiopia, 2017

**DOI:** 10.1155/2021/9927779

**Published:** 2021-11-08

**Authors:** Tadeg Jemere, Berhanu Getahun, Mahlet Tesfaye, Geremew Muleta, Nega Yimer

**Affiliations:** ^1^Department of Biomedical Sciences, College of Health Sciences, Debre Tabor University, Debre Tabor, Ethiopia; ^2^Integrated Emergency Gyn/Obs and General Surgery Professional, Amedwerk Hospital, Amdewerk, Ethiopia; ^3^Department of Surgery, School of Medicine, College of Medicine and Health Science, Jimma University, Jimma, Ethiopia; ^4^Department of Public Health, College of Medicine and Health Science, Jimma University, Jimma, Ethiopia; ^5^Department of Nursing, College of Medicine and Health Sciences, Wollo University, Dessie, Ethiopia

## Abstract

**Background:**

Small bowel obstruction is a common and dangerous surgical emergency which is associated with high morbidity and mortality if not managed appropriately and timely.

**Objective:**

To determine the causes and management outcome of small bowel obstruction in Nekemte Referral Hospital, Nekemte, Ethiopia.

**Method:**

Institution-based retrospective cross-sectional study design was used. Three-year data (from January 1, 2014, to December 30, 2016) were collected from July 1 to August 30, 2017. Data were collected from medical records and checked for any inconsistency, coded, and entered into SPSS version 20 for analysis. Descriptive, binary, and multivariate logistic regression analyses were used. On binary logistic regression analysis, variables with *p* ≤ 0.25 were selected as a candidate for multivariate logistic regression analysis. The level of statistical significance was set at *p* ≤ 0.05.

**Results:**

With 100% response rate, records of 211 patients with small intestinal obstruction were retrieved for analysis. One hundred thirty-seven (64.9%) were males. The commonest cause of small bowel obstruction was adhesion (35.1%). More than a quarter (26.5%) participants developed postoperative complications, and wound infection was the commonest postoperative complication (49.2%). A majority (84.8%) of patients improved and were discharged, and the rest 15.2% of patients died. Sex (AOR = 3.98, 95% CI: 1.51–10.52), duration of illness before surgical intervention (AOR = 4.4, 95% CI: 1.69–11.45), level of hematocrit (AOR = 4.25, 95% CI: 1.56–11.57), types of intestinal obstruction (AOR = 3.73, 95% CI: 1.09–12.64), and length of hospital stay (AOR = 4.69, 95% CI: 1.82–12.07) were independent predictors of the management outcome of patients with small bowl obstruction.

**Conclusion:**

Small bowel obstruction is a commonly encountered surgical emergency. Adhesion, small bowel volvulus, and intussusception were the leading causes of small bowel obstruction, respectively.

## 1. Introduction

Small bowel obstruction (SBO) is defined as blockage of the passage of small intestinal contents from the proximal to distal segment. It is one of the most common conditions resulting in hospital admissions [1].

Small bowel obstruction is a common and dangerous surgical emergency which is associated with high morbidity and mortality if not managed appropriately and timely. The prevalence and causes of SBO differ internationally and locally [2–5], but it is a serious surgical emergency worldwide, with increased rate of morbidity and mortality. The situation was considered to be worse in developing countries where health facilities were rare and health education was lacking, and numerous patients who present late to hospitals after trials with local remedies were exhausted. Across the world, there is variation in the pattern of mechanical bowel obstruction [6, 7].

The most common risk factor for adhesive obstruction is violation of the peritoneal cavity following laparotomy. It is possible that talc or starch of the surgical gloves in routine use in our environment played a role in adhesion formation in some of the patients [4].

Primary small bowel volvulus is one of the other common causes of SBO in parts of Africa. It was more common during the rainy seasons, that is, through June to October [5, 6].

The pattern of the disease changes from time to time and needs periodic studies to evaluate the causes and prevalence of the disease. The causes of SBO are several, and their relative incidence varies across different populations and countries. It has also shown variation over the decades [7]. The knowledge of patterns and causes of SBO in a country as well as in different parts of the country has a significant value in fast diagnosis and on timely surgical intervention, which results in good outcome of the patient [8–10].

The management outcome of the diseases may be a good indicator of how well a country's surgical services are doing. Several factors contribute to the poor outcome of patients with SBO. Some of these factors may include poor health-seeking behavior, ignorance, poverty, and poor clinical judgment [2, 3]. Thus, the main aim of this study was to determine causes and management outcome of small intestinal obstruction in Nekemte Referral Hospital (NRH).

## 2. Methods

### 2.1. Study Setting and Period

A hospital-based retrospective cross-sectional study was conducted at NRH, Nekemte town. Nekemte town is located in Oromia regional state, western Ethiopia, which is 333 kilometers from Addis Ababa. The hospital was established in 1923 EC, providing service for approximately 3 million people in the catchment area including surgery. The study was conducted from July 1 to August 30/2017.

### 2.2. Source and Study Population

All patients admitted to NRH and managed for bowel obstruction were source populations. All patients admitted with a diagnosis of small intestinal obstruction at NRH from January 1, 2014, to December 30, 2016, were study populations.

### 2.3. Eligibility Criteria

#### 2.3.1. Inclusion Criteria

- Patients who were clinically diagnosed with SBO and managed conservatively without operation
- Patients who were clinically diagnosed with SBO and managed operatively
- Patients who died after presenting with the clinical diagnosis of small intestinal obstruction without surgery
- Patients with mechanical and nonmechanical (adynamic) intestinal obstruction

#### 2.3.2. Exclusion Criteria

- Incompletely documented charts
- Patients whose charts were lost

### 2.4. Sample Size Determination and Sampling Technique

All 211 patients admitted to the surgical ward of NRH with the diagnosis of SBO and treated from January 1, 2014, to December 30, 2016, were included.

### 2.5. Operational Definitions

Favorable management outcome: if patients did not develop either postoperative complication or death after conservative or operative management.

Unfavorable management outcome: if the patient developed one or more postoperative complications and/or death.

### 2.6. Data Collection Procedure

The checklist was first developed by English language and translated to Amharic language and then retranslated back to English to maintain its consistency. Patients that were admitted to the surgical ward of NRH with the diagnosis of SBO and treated were initially identified from admission logbooks of surgical wards and operation theater of NRH from which the chart number of patients was obtained. Then, charts of the patients were retrieved from the card room. Relevant information was collected from these charts.

### 2.7. Data Analysis Procedure

The collected data were coded and entered into SPSS version 20 for data analysis. Association between the management outcome of SBO and independent variables was checked by using the binary and multivariate logistic regression. On binary logistic regression, a *p* value ≤0.25 was used as a candidate for multivariate logistic regression analysis. A statistical significant association was tested at a *p* value <0.05.

### 2.8. Data Quality Management

Training for data collectors was given for 2 days regarding the purpose of the study, on how to fill the prepared checklist, and the importance of data quality. In order to avoid interpersonal variation between data collectors, data were collected by the same data collectors throughout the study period. Regular daily supervision was conducted to check the consistency and completeness of the checklists.

## 3. Results

### 3.1. Sociodemographic Characteristics

A total of 301 patients were admitted and managed for intestinal obstruction from January 1, 2014, to December 30, 2016, and of whom, 211 (70.7%) were SBO. From the total SBO cases, 41.7% were aged 21–44 years with a mean age of 27.2 (SD ± 19.2). From the total participants, 64.9%, 34.6%, 24.2%, and 68.7% were males, illiterates, farmers, and from rural area, respectively (Table 1).

### 3.2. General Condition of the Patient

From a total of patients participated, 144 (68.2%) came within 24 hours of manifestation, and 159 (75.4%) of the patients were with hematocrit of >36%. In addition, 113 (53.6%) of participants came with referral, and 78 (37%) had previous abdominal operation. Eighty-one (38.4%) of the participants were with preoperative complication.

### 3.3. Causes of Small Bowel Obstruction

In cases managed during the study period, dynamic bowel obstruction was the leading cause (90.5%), and adhesion, SBV, and intussusceptions (74 (35.1%), 51 (24.2%), and 50 (23.7%)) were the commonest intraoperative findings, respectively (Table 2).

### 3.4. Management Outcome

Laparotomy was the most common method of small intestinal obstruction management in this study (95%), and 5% were improved by conservative management. Bowel resection and anastomosis were the commonest intraoperative procedures done. From a total of operated cases, 26.5% developed postoperative complication with SSI being the most common complication (49.2%), 61.6% stayed in the hospital for less than seven days, and 84.8% were improved and discharged.

### 3.5. Factors Associated with the Management Outcome of Small Bowel Obstruction

A total of ten variables (age, sex, educational status, duration of illness, hematocrit level, previous history of abdominal operation, preoperative complication, type of obstruction, intraoperative findings, and length of hospital stay) were found to have an association with the management outcome of SBO on bivariate analysis.

### 3.6. Predictors of the Management Outcome of Small Bowel Obstruction

In the multivariable regression model, male sex (AOR = 3.98, 95% CI: 1.51–10.52), duration of illness before surgical intervention (AOR = 4.4, 95% CI: 1.69–11.45), level of hematocrit (AOR = 4.25, 95% CI: 1.56–11.57), types of intestinal obstruction (AOR = 3.73, 95% CI: 1.09–12.64), and length of hospital stay (AOR = 4.69, 95% CI: 1.82–12.07) were significantly associated with the management outcome of small bowel obstruction (Table 3).

## 4. Discussion

Small bowel obstruction is a commonly encountered emergency condition in hospitals worldwide and a leading cause of emergency room visits. This postures a challenge to the surgical trainee. Its treatment requires cautious preoperative preparation, great surgical judgment, and postoperative care which are frequently very demanding [3, 4]. This hospital-based study has tried to address causes and the management outcome of small intestinal obstruction in Nekemte Referral Hospital.

The study shows small intestinal obstruction is more prevalent in males (62.1%) than in females (37.9%). This is comparable with other studies conducted in Larkana, Rwanda, Nigeria, and southern Ethiopia [3, 7, 8].

In this study, duration of obstruction before surgical intervention has a statistical significant association with the management outcome of patients. Patients who presented after 24-hour duration of obstruction are 4.4 times more likely to have unfavorable result as compared with patients who came to the hospital within 24 hours. This is in agreement with the studies conducted in Rwanda, Adama, Ethiopia, and Niger [3, 9, 11].

With early diagnosis and suitable management, most of patients enduring from intestinal obstruction can be spared. Most of the time, the situation is quite different. Some patients come after subjecting themselves to relatively long periods of observation, and they usually present to us when they really feel very sick [3]. In this study, 64.9% of patients presented within 24-hour duration of illness, while 35.1% presented after 24 hours. This is in line with the studies done in Pakistan, Rwanda, and Nigeria [3, 5, 7]. Delayed presentation and/or surgical intervention frequently results in unfavorable surgical outcome and/or longer hospital stay. Reasons for delay may include low socioeconomic status, poor infrastructure, and low health-seeking behavior.

This study also shows that patients who stayed in the hospital for shorter than 7 days after surgery were about 5 times more likely to have favorable outcomes when compared with those who stayed for longer than or equal to 7 days after the surgery. This is similar with the result of studies conducted in Ethiopia [6, 9, 10] and Uganda [12]. Long period of hospital stay may increase the chance of acquiring nosocomial infections, and this may contribute to the poor outcome of patients with long hospital stay.

In this study, lower hematocrit level was associated with an increased occurrence of unfavorable outcomes. Participants with hematocrit greater than or equal to 36% were 4.25 times more likely to have favorable outcomes when compared with those who had hematocrit less than 36%. It is in line with the study conducted in Ethiopia [6]. In addition, participants with dynamic obstruction were around four times more likely to have favorable outcomes when compared to adynamic obstruction. It is consistent with the study conducted in Adama, Ethiopia [10].

The most frequently occurred postoperative complications were wound infection (49.1%), which is consistent with other studies conducted in Gondar, Adama, and Sudan [9, 10, 13]. Most of the postoperative complications were with cases who present late and those who were with preoperative complications and with the hematocrit level of less than 36%.

One hundred seventy-nine (84.8%) patients improved and were discharged after they obtained service in the hospital, whereas 32 (15.2%) died after being operated, and the result is comparable with studies done in Gondar (13.3%), Sudan (15.7%), Nigeria (14%), and Tanzania (14.3%), respectively [8, 13–15], but studies done in Larkana, Pakistan, and Iraq show an overall mortality rate of 6.6%, 3.49%, and 4.3%, respectively [1, 5, 16]. This difference in mortality rate might be associated with late presentation of patients to the hospital due to the lack of awareness about the burden and impacts of the disease, preoperative complications, and the level of adequate preoperative resuscitation between the countries.

Adhesions are the single most common cause for small bowel obstruction [17]. Our study also supports this as it was the most common intraoperative finding (35.1%), followed by SBV (24.2%) and intussusceptions (23.7%). This is in agreement with the result of the studies conducted in India [18] and Italy [19].

### 4.1. Limitation of the Study

The limitation of this study was that secondary data were used.

## 5. Conclusion

Adhesion, small bowel volvulus, and intussusceptions were the main causes of small bowel obstruction. Laparotomy was the most common means of intestinal obstruction management, and bowel resection and anastomosis were the commonest intraoperative procedures done. The most frequently occurred postoperative complication was wound infection. Sex, duration of illness before surgical intervention, hematocrit level, types of intestinal obstruction, and period of hospital stay were predictors of the management outcome of patients with small bowl obstruction.

## Figures and Tables

**Table 1:** Sociodemographic characteristics of patients managed for small bowel obstruction in Nekemte Referral Hospital from January 1, 2014, to December 30, 2016.

Variables		Frequency (*N* = 211)	Percentage
Age (years)	≤20	60	28.4
	21–44	88	41.7
	≥45	63	29.9

Sex	Female	74	35.1
	Male	137	64.9

Educational status	Illiterate	73	34.6
	1–8	47	22.3
	9–12	15	7.1
	College and above	30	14.2
	Others (preschool children)	46	21.8

Occupation	Farmer	51	24.2
	Merchant	9	4.3
	Employee	27	12.8
	Student	37	17.5
	House wife	41	19.4
	Others (preschool children)	46	21.8

Residence	Rural	145	68.7
	Urban	66	31.3

Distance from the hospital (km)	<50	121	57.3
	≥50	90	42.7

**Table 2:** Causes and management approaches of patients managed for small bowel obstruction in Nekemte Referral Hospital from January 1, 2014, to December 30, 2016.

Variables		Frequency (*N* = 211)	Percentage
Type of intestinal obstruction	Dynamic	191	90.5
	Adynamic	20	9.5

Preoperative anticipated cause of obstruction	Adhesion	78	37.0
	Volvulus	62	29.4
	Intussusception	50	23.7
	Others (unspecified SBO, ISK, and mesenteric ischemia)	21	10.0

Mode of patient management	Laparotomy	203	96.2
	Conservative	8	3.8

If managed by laparotomy, what was the intraoperative finding?	Adhesion	74	35.1
	SBV	51	24.2
	Intussusception	50	23.7
	Herniated small bowel	28	13.3
	Others	8	3.8

**Table 3:** Predictors of the management outcomes of small intestinal obstructions in Nekemte Referral Hospital from January 1, 2014, to December 30, 2016.

Variables		Unfavorable	Favorable	COR (95% CI)	*p* value	AOR (95% CI)
Age (years)	≤20	10	50	1		
	21–44	9	79	1.76 (0.67–4.620)	0.86	1.48 (0.48–4.53)
	≥45	13	50	77 (0.31–1.92)	0.94	1.32 (0.41–4.22)

Sex	Female	20	54	1		
	Male	12	125	3.86 (1.76–8.45)	0.001^∗^	3.98 (1.51–10.52)1^∗^

Duration of illness before presentation (hours)	≥24	21	46	1		
	<24	11	133	5.52 (2.47–12.32)	<0.00011^∗^	4.4(1.69–11.45)1^∗^

Hematocrit (%)	<36	16	36	1		
	≥36	16	143	3.97 (1.82–8.69)	0.0011^∗^	4.25 (1.56–11.57)1^∗^

Type of obstruction	Adynamic	8	12	1		
	Dynamic	24	167	4.64 (1.72–12.51)	0.0021^∗^	3.73 (1.09–12.64)1^∗^

Hospital stay (days)	>7	21	60	1		
	≤7	11	119	3.79 (1.71–8.37)	0.0011^∗^	4.69 (1.82–12.07)1^∗^

^∗^Statistically significant.

## Data Availability

The authors confirm that all the data underlying the findings are fully available without restrictions. All relevant data are included within the article.

## Abbreviations

AOR: Adjusted odds ratio
BMI: Body mass index
CI: Confidence interval
STI: Sexually transmitted infection.
